# Considerations in surgical versus non-surgical management of HPV positive oropharyngeal cancer

**DOI:** 10.1186/s41199-016-0007-8

**Published:** 2016-07-11

**Authors:** Christopher E. Fundakowski, Miriam Lango

**Affiliations:** Fox Chase Cancer Center, 333 Cottman Ave, Philadelphia, PA 19111 USA

**Keywords:** Human Papilloma Virus, Neck Dissection, Intensity Modulate Radiation Therapy, Tongue Base, Extracapsular Extension

## Abstract

Given the marked difference in clinical presentation and treatment response based on human papilloma virus (HPV) status, HPV-associated oropharyngeal squamous cell carcinoma is now viewed as a distinct biologic and clinical entity. HPV-associated oropharyngeal squamous cell carcinoma has increased by nearly 7.5 % per year, from approximately 16 % in the early 1980′s to nearly 70 % today, and is believed will continue to increase dramatically in the coming years. Currently, a myriad of treatment options exist for these patients as many active clinical trials are underway which aim to identify the most appropriate interventions for this unique group of patients. This review aims to provide considerations between surgical and non-surgical management for HPV-associated oropharyngeal squamous cell carcinoma.

## Background

Pharyngeal carcinoma incidence worldwide is approximately 136,000 [1]. Between 1983 and 2002, an examination of 23 countries across four continents noted a significant rise in oropharyngeal squamous cell carcinoma (OPSCCA), particularly in young men in economically developed countries [2]. There are now approximately 13,000 new cases diagnosed annually. Historically, as with other mucosal head and neck subsites, etiology of OPSCCA was thought to be primarily secondary to tobacco and alcohol use. We now know human papilloma virus (HPV) to be a major driver of OPSCCA. While the overall incidence of HPV-negative OPSCCA decreased alongside declining smoking rates between 1988 and 2004, HPV-associated OPSCCA has increased by nearly 7.5 % per year, from approximately 16 % of all OPSCCA in the early 1980′s to nearly 70 % today, and is believed will continue to increase dramatically in the coming years [3–8].

The presence of HPV has served as a significant prognosticator in terms of survival in OPSCCA, first reported in the late 1990′s [4, 6, 9]. Excellent treatment response rates (>80 %) correspond with the improved survival, and are preserved regardless of specific treatment modality [10]. Differences in three-year progression free survival (which includes subset analysis of RTOG 0129) between HPV-associated and HPV-negative OPSCCA are impressive at 75–82 % and 45–57 %, respectively, with HPV-associated outcomes improving over time, and HPV-negative outcomes remaining stable, and poor [3–5, 8, 11–14]. Locoregional failure at three years is also significantly lower in the HPV-associated patient compared to HPV-negative, at 13.6 % VS 35 %, respectively; these differences in survival and failure have been confirmed independently and prospectively [4]. HPV associated in the context of OPSCCA affords a 51 % lower risk of disease progression, and 58 % lower risk of death. Interestingly, the negative impact of tobacco use on prognosis remains in these patients and is independent of HPV status [3]. Regarding recurrences, the majority occur within the first year after treatment and are locoregional [15]. While the rate of locoregional failure in HPV-associated OPSCCA is lower, the rates of distant metastasis are similar to HPV-negative OPSCCA, with the lung being the most common site of failure [15]. Fortunately, even in the context of recurrent and/or metastatic disease, HPV association serves as a favorable prognosticator, and consequently aggressive treatment in both these settings may remain justified.

In addition to survival and failure differences, marked differences in clinical presentation characteristics have been noted. Historically, the “typical” head and neck cancer (HNC) patient is of advanced age and with significant substance abuse history. Today, the HPV-associated OPSCCA patient is noted to be younger (between 4th and 6th decade), male (8:1, M:F), with no/minor smoking history, predominantly Caucasian, increased education, married, and higher income [8]. In terms of exam characteristics, HPV-associated lesions commonly present with comparatively smaller primary tumor (less than 4 cm), and comparatively more advanced nodal disease, which leads to a high TNM and overall stage which may overestimate disease severity as it relates to other head and neck cancers [16, 17].

Given the marked difference in clinical presentation and treatment response based on HPV status, HPV-associated OPSCCA is now viewed as a distinct biologic and clinical entity. Consequently, novel staging systems have been proposed and validated for the staging of HPV-associated OPSCCA to better represent disease severity/prognosis, though none formally adopted at this time [18]. In addition, current treatment guidelines do not account for or recommend modifications based on HPV status, but likely will in the future. Several clinical trials are enrolling HPV-associated OPSCCA patients, assessing surgical intervention, radiation de-escalation, and alterations in systemic therapy. These carefully planned de-intensification strategies focus on treatment modifications which will preserve disease control and minimize both long and short term morbidity in selected groups of OPSCCA patients based on T stage, N stage, and smoking status [19–21].

This review aims to provide considerations between management strategies for HPV-associated OPSCCA, noting that at the current time, curative treatment for many of these patients outside the context of clinical trial may include multiple modalities. While no level 1 data exists which provides a comparison between surgical and non-surgical approaches in HPV-associated OPSCCA, and previous retrospective studies are with expected limitations and selection bias, we will discuss clinical considerations and decision-making associated with both of these approaches.

## Treatment modalities

Treatment options for HPV-associated OPSCCA may be largely divided into radiotherapy, systemic therapy/chemotherapy, and surgical resection. Depending on stage and extent/presence of adverse features, definitive treatment may include a combination of modalities.

### Radiation therapy

Intensity modulated radiation therapy (IMRT) has been instrumental in preserving excellent control rates, while improving on treatment-related toxicity, with particular regards to salivary and pharyngeal structures [22, 23]. For stage 1/2 OPSCCA, IMRT notes impressive two year disease free survival between 82 and 90 %, with two year disease specific survival up to 97 %. Regarding toxicity, rates of xerostomia – 18 % (grade 2); dysphagia – 15 % (grade 2); subclinical aspiration – up to 50 % (though reported incidence of aspiration pneumonia much less at approximately 14 %); hypothyroidism is noted in 28–38 % of patients at three years, though may be as high as 55 % depending on the gland volume and amount of gland receiving over 45 Gy; esophageal stenosis -approximately 5 %; osteoradionecrosis of the mandible - approximately 2.5 %; and need for gastrostomy tube to be placed at some point during or up to one year after treatment is around 4 %, though with prolonged followup, may increase to 16 % [23–25].

Current dosing strategies are largely based on randomized trials which lack predominant HPV-associated OPSCCA cohorts. As a result, the optimal treatment and dosing strategy is still in the process of being determined. Unilateral neck radiation for lateralized tonsil cancer is warranted, while bilateral neck radiation is commonplace for tongue base primaries. The potential for unilateral neck radiation in the context of lateralized tongue base cancer is one for debate at this time. Regarding delivery, neither RTOG 9003 nor RTOG 0129 revealed altered fractionation to deliver a survival advantage over standard once-daily fractionation [26, 27].

Currently, guideline dosing recommendations have not been altered based on HPV status, nor recommended outside the context of a clinical trial. Retrospective analysis of node positive HPV-associated OPSCCA noted a 96 % five year regional control after dose de-escalated radiotherapy (54 Gy), with 90 % receiving concurrent cisplatin based chemotherapy [28]. A prospective trial (ECOG 1308) also de-intensified to 54Gy with excellent locoregional control [29]. Radiation dose de-escalation is a major area of interest since short and particularly long term toxicities are largely attributable to radiation dose. Many patients with HPV-associated OPSCCA will survive 30-plus years post-treatment and thus have increased opportunity to fall victim to long-term side effects.

While radiation is commonly utilized concurrently with chemotherapy, radiation monotherapy is a potential effective modality as well and is typically reserved for early stage HPV-associated OPSCCA (T1-T2, N0-1). Indications for radiation monotherapy may be expanded (to include larger T stage and higher N stage) based on results of ongoing trials, such as RTOG 1333 (NRG-HN002) which compares radiotherapy alone versus concurrent chemoradiotherapy (with reduced dose cisplatin, 40 mg/m^2^) in p16 positive locally advanced OPSCCA patients with minor/no smoking history (10 or less pack years).

### Chemotherapy

Much of the data utilized in clinical decision making for HPV-associated OPSCCA is extrapolated from advanced HNC trials not specifically focused on HPV status. High-dose cisplatin is considered the standard agent for concurrent therapy in HNSCCA. The Intergroup trial of patients with locally advanced HNC noted greatest survival to be in the treatment group of those who received concurrent radiation and cisplatin [30]. However, robust prospective comparative studies with alternative agents have not been completed. Cetuximab, a monoclonal antibody which targets epidermal growth factor inhibitor, is an agent of interest used quite widely in HNC. Bonner et al. demonstrated a 10 % survival benefit at three years in advanced stage HNC when cetuximab was administered concurrently with radiation [31]. Side effect profile is comparatively favorable, with the exception of acneiform rash, cetuximab does not have an association with increased grade 3/grade 4 toxicities. Unfortunately, to date no clinical trial data is available directly comparing cetuximab vs cisplatin for HPV-associated oropharynx cancer, but RTOG 1016 which has completed primary accrual should provide evidence to address this question.

Regarding advanced non-HPV-related HNC, the addition of cytotoxic chemotherapy is noted to improve survival over radiation alone, by enhancing locoregional control [32, 33]. When utilized as initial definitive treatment in the context of advanced OPSCCA, concurrent chemoradiation was noted to be superior to induction chemotherapy followed by radiation [32].

Chemotherapy also is utilized alongside radiation in the setting of high risk/adverse features (extracapsular extension, positive/close margin) with improved disease free survival and locoregional control noted in combined treatment arm of European Organization for Research and Treatment of Cancer (EORTC) 22931 and RTOG 9501 [34, 35]. An improvement in locoregional control and survival of adjuvant chemoradiation has not been observed for patients with HPV-associated OPSCCA and extracapsular extension of metastatic cervical lymphadenopathy [36]. A review by Lewis et al described the lack of impact of extracapsular spread on survival as well as a lack of association between size of nodal metastasis and extracapsular spread [37]. Sinha et al. assessed outcomes in HPV-associated SCCA with nodal extracapsular spread and no survival advantage was noted with the addition of chemotherapy to radiotherapy [38]. However, the sample size required to detect a survival difference is very large given the paucity of events, and the favorable survival of patients with HPV-related OPSCCA. It is likely that prior studies were underpowered to detect an effect of chemotherapy on survival. Currently, the postoperative adjuvant therapy de-intensification trial for HPV-related OPSCCA (ADEPT trial, phase III randomized) aims to definitively answer the question of the utility of chemotherapy for these patients (assessing two year locoregional control and disease free survival), though the estimated primary completion date is October 2018.

The use of concurrent chemoradiation for locoregionally advanced HNC carries with it increased acute and late toxicities, over radiation alone or surgery. While the GORTEC trial noted concurrent chemoradiation to provide superior overall survival and locoregional control compared to radiation alone, it resulted in increased late complications, increased need for gastrostomy tube, and mucositis [39]. Randomized clinical trials report chemoradiation to have a treatment-related mortality rate of approximately 3–4 % [40].

There is opportunity to further define the role of chemotherapy in HPV-associated OPSCCA, as this group of patients generally has a favorable prognosis and the question remains if the treatment related toxicities of concurrent chemoradiation provide meaningful benefit in terms of disease control and survival.

### Surgical approaches

Historically, adequate access for many oropharyngeal tumors required a transcervical approach +/-mandibulotomy. With improvements in both imaging and instrumentation, transoral surgery has become much more widespread. When referring to NCCN guidelines, it is offered as an equivalent option (to open surgery) in all treatment algorithms. However, the safety, efficacy and tolerability of modern, minimally invasive transoral approaches suggest that transoral procedures should be considered over traditional transcervical approaches for properly selected patients.. We will limit discussion mainly to transoral approaches, which include transoral robotic surgery (TORS) and transoral laser microsurgery (TLM) which represent two different minimally invasive techniques. To date, no formal trial has tested TORS vs TLM in a head to head fashion. Clinical trials that include minimally invasive transoral surgery (such as ECOG 3311) leave the decision to use TORS or TLM at the discretion of the surgeon, and neither specify criteria for patient selection. Both TLM and TORS are well-established techniques with proven oncologic outcomes [41–43]. Both require specialized equipment and associated expense, training/credentialing, and are associated with a learning curve. A significant advantage of TORS over TLM includes the use of angled telescopes and rotating robotic surgical arms which obviate some limitations of surgery dependent on the line of sight.

Compared to traditional approaches, both TORS and TLM are less disruptive of normal and uninvolved anatomic structures, and consequently, the use of these approaches has been associated, with a shorter hospital stay, faster recovery, less pain, less need for gastrostomy/tracheostomy tube, and has less cost. [44] The immediate postoperative morbidity can be substantial, and varies with the extent of the resection. Yet, minimally invasive surgical treatment yields far less long-term sequelae than classic transcervical surgery; in the absence of postoperative radiation, or chemoradiation, minimally invasive transoral surgery yields minimal late sequelae [45].

Oncologic outcomes following minimally invasive surgery have similarly been favorable. In 2012, a review of TORS outcomes noted a two year overall survival of 80–90 % for a cohort consisting of 36 T1/2, 11 T3/4 with neck disease ranging from N0-N2c [46]. TLM results have noted five year overall survival of 78 %, with local control rates of 85–97 % [47, 48]. TORS/TLM is not only limited to early stage cancers, as several series with advanced stage OPSCCA have noted up to 90 % local control and disease specific survival [41, 48]. Interestingly in these series, between 38 and 84 % of patients avoided chemotherapy. In a series by Haughey et al., postoperative swallowing results were excellent in 87 % of patients; long-term dysphagia was associated with T4 tumors though, particularly those which involved the tongue base [48]. As one may expect, postoperative functional outcomes were noted to further worsen with the addition of each adjuvant modality.

Lacking high-level data to support oncologic superiority of surgery over non-surgical approaches, the decision to employ a surgical approach is driven primarily by the need for adequate surgical exposure as well as functional and quality of life considerations. Patient selection is extremely important as transoral surgery should be utilized for OPSCCA which is adequately visualized and anatomically favorable for complete resection, thus enabling the patient to avoid potentially unnecessary adjuvant therapy. Non-surgical treatment with radiation or chemoradiation remains a mainstay of treatment, if surgical intervention is not favorable. Tumor characteristics which will commonly dissuade from primary surgical intervention include: tongue base invasion which will require greater than 50 % resection, pterygoid muscle involvement, extension into the parapharyngeal fat abutting the carotid, mandible/maxilla extension or invasion, and prevertebral space involvement.

The adequacy of the surgical extirpation has a great impact on the delivery of postoperative radiation. With a positive margin, most radiation oncologists will deliver a full dose of radiation to the primary site, with concurrent chemotherapy. A negative margin warrants a lower radiation dose, and possibly a reduction in the target volume. Given the bulk of some tumors within the oropharynx, uninvolved pharyngeal structures immediately adjacent to the mass may have a higher radiation exposure. The ability to perform radiation planning in the absence of that tumor bulk may allow the radiation oncologist to tailor the treatment volume to the area of interest potentially yielding improved swallowing outcomes [49].

Surgical management of the neck varies by location of the primary and the presenting N stage. Neck dissection may be performed at the time of primary tumor resection, or may be staged. There may be for various reasons for staging of the neck dissection, such as a concern for communication between the oropharynx and the neck, or for reasons of equipment logistics (time constraints of the “robot” room for example). For tonsil/lateral pharyngeal wall-based primaries, dissection of the N0 neck will typically involve levels 2–4 ipsilaterally. For N+ necks, nodal dissection will be adjusted based on positive node location and size. In the context of tongue base primaries which approach/cross the midline, bilateral neck dissection is indicated, particularly when considering using surgery as a single curative modality.

An additional benefit of primary surgical treatment for OPSCCA is the ability to have definitive pathology (such as margin status and presence/degree of extranodal extension) from both tumor and cervical nodes for staging and subsequent decision-making purposes regarding adjuvant therapy. This may be of benefit over clinical/radiographic staging given the potential for over/under-staging. In a study by Walvekar et al. involving nearly 50 patients with OPSCCA who underwent primary surgical resection, 40 % of patients had their preoperative staging changed, 24 % of which were down-staged [50]. The ability to accurately stage and offer subsequent therapeutic de-intensification is of great importance in terms of clinical trials for OPSCCA and for future tailored therapy.

## Clinical decision-making

Ideally optimal treatment will provide maximum survival opportunity while minimizing locoregional recurrence and distant failure [51]. In HPV-associated OPSCCA, single modality therapy should be utilized when appropriate; the avoidance of triple modality therapy is recommended to minimize toxicity. The clinical expertise/experience of the treatment team cannot be underestimated with regards to maximizing disease control and minimizing morbidity [52]. Once the patient has been staged appropriately, how does one decide between surgical vs non-surgical management?

### Survival and disease control

One of the first considerations for both clinician and patient will be regarding survival and disease control. When comparing transoral resection followed by adjuvant treatment (as dictated by NCCN guidelines) to nonsurgical management, the results of local control, regional control, and survival are comparable [53, 54]. As a result, attention to treatment-related morbidity, functional outcome, and quality of life is of great importance, and commonly the main driver behind treatment approach.

### Patient factors

Multi-disciplinary evaluation is the foundation of comprehensive care of the cancer patient. Pretreatment assessment of baseline functionality is critically important. An evaluation of anesthesia risk, oral/oropharyngeal function, swallowing and airway protection, along with potential for rehabilitation is essential. After appropriate pre-treatment counseling, patient preference in terms of surgical vs non-surgical management must also be considered.

The patient smoking history is also taken into consideration, as we know this to be an independent prognosticator when greater than 10 pack/years [3, 55]. Interestingly, epidemiologic data describes smoking as an independent risk factor for the development of OPSCCA in general, but not specifically the development of HPV-associated OPSCCA [56]. Ang et al note though that the biologic behavior of HPV-associated OPSCCA is likely altered by tobacco use, with decreased responsiveness to therapy [3]. As a result, smoking serves as an independent risk factor for overall survival and progression free survival in HPV associated OPSCCA in multivariable analysis. The negative effect on survival increases as pack-years of smoking increases [55]. Ang et al. utilized a 10 pack-year cut off to designate a low risk group, as it was noted to be the best survival predictor in recursive partitioning analysis. The pack-year cutoff was also elected over a continuum for ease of future trial design, acknowledging the need for further validation.

Current de-escalation protocols consider patients to be in the lowest risk smoking category when at 10 or less pack-years. Specific situations may exist though which still consider a patient to be low-risk despite a greater than 10 pack-year history, such as in ECOG 3311, with a patient possessing a T1-2, N0-1 tumor with negative margins and no extracapsular extension. RTOG 1333 (NRG-HN002) uses a cut-off of 10 pack years as well. Of interest, the low-risk group in the investigation by Ang et al. also contained patients with greater than 10 pack-year, though limited to N0-N2a; so it remains to be seen whether the de-escalation envelope will be pushed in the future for those with higher than 10 pack-year smoking history if they possess limited neck disease. See Table 1 for additional trials related to HPV-associated OPSCCA.

**Table 1:** De-escalation trials in HPV-associated OPSCCA

Trial	Phase	Population	Intervention	Primary outcome
Surgical trials				
NCT01898494 ECOG 3311	II	Stage III-Iva (no T1-2, N1)	Transoral surgery followed by risk stratification -*Low*: no adjuvant -*Intermediate*: 50 vs 60 Gy IMRT randomization -*High*: 66 Gy IMRT with weekly cisplatin (40 mg/m2)	2 year DFS
NCT01932697	II	<10 pack year smoker, must have one: T3/T4a, N2a-N3, ECE/PNI/LVI	Transoral surgery with negative margin followed by hyperfractionated IMRT + docetaxel	2 year LRC/DFS
NCT01687413, ADEPT	III	T1-4a with negative margins and cervical metastasis with ECE	Transoral surgery followed by IMRT: randomized into 60 Gy alone vs with concurrent cisplatin (40 mg/m2)	2 year DFS/LRC
NCT02215265, PATHOS	II/III	T1-3, N0-2b, <10 pack year smoker	Transoral surgery followed by risk stratification -*Low*: no adjuvant -Intermediate: 50 vs 60 Gy IMRT randomization *-High*: chemoradiation vs IMRT (60 Gy) randomization	2 year PFS
NCT01590355, ORATOR	II	T1-2, N0-1 or N2b (up to 2 nodes); stratify groups by HPV status	Transoral surgery plus neck dissection vs IMRT +/- chemotherapy	1 year QOL
EORTC 1420-HNCG-ROG	III	Stage I/II, HPV positive and negative	Transoral surgery plus neck dissection randomized vs IMRT	1 year QOL
NCT02072148, SIRS	II	T1N0-2b, T2N0-2b; <20 pack year smoker (or >10 year smoke free)	Transoral surgery followed by risk stratification -*Low*: no adjuvant -Intermediate: 50 Gy IMRT *-High*: IMRT (56 Gy) + cisplatin (40 mg/m2)	3 & 5 year DFS/LRC
Non-surgical trials				
NCT01084083, ECOG 1308	II	Stage III/IVa-b	Low dose IMRT (50 Gy) + cetuximab vs standard dose (60 Gy)	2 year OS
NCT02254278, NRG HN002	II	Stage III/IV (T1-2, N1-2b, or T3, N0-2b); <10 pack year smoker	IMRT (60 Gy) +/- cisplatin	2 year PFS
NCT01302834, RTOG 1016	III	Stage III/IV	IMRT (60 Gy) with high dose cisplatin vs IMRT with cetuximab	5 year OS
NCT01874171, De-ESCALATE	III	T3N0-T4N0 and T1N1-T4N3), <10 pack year smoker	Standard dose IMRT (70 Gy) with cisplatin (100 mg/m2) vs IMRT + cetuximab	Severe toxicity
NCT01855451, TROG 12.01	III	Stage III (except T1-2 N1), stage IVa (except T4), <10 pack year smoker (if >10 pack year, must be N0-N2a)	Standard dose radiation (70 Gy) with cisplatin (40 mg/m2) vs cetuximab	Symptom severity
NCT01663259, University of Michigan	II	Stage III/IV (no T4,N3), <10 pack year smoker	Standard dose radiation (70 Gy) + cetuximab	Recurrence rate
NCT01530997, Lineberger Comprehensive Cancer Center	II	T1-3, N0-2c; <10 pack year smoker (or > 5 years smoke free)	IMRT (54-60 Gy) with cisplatin (30 mg/m2)	Complete pathologic response
NCT01088802, Kimmel Comprehensive Cancer Center	II	T1-3, any N (resectable)	IMRT, dose de-escalation from 70 Gy to 63 Gy, and from 58.1 Gy to 50.75 Gy, while receiving cisplatin	Severe toxicity, QOL

Abbreviations: *ECOG* Eastern cooperative oncology group, *ADEPT* adjuvant de-escalation, extracapsular spread, p16-positive, transoral, *ECE* extracapsular extension, *DFS* disease-free survival, *LRC* loco-regional control, *HPV* human papilloma virus, *OPSCCA* oropharyngeal squamous cell carcinoma, *PATHOS* postoperative adjuvant treatment for HPV-positive tumors, *PFS* progression free survival, *IMRT* intensity modulated radiation therapy, *ORATOR* oropharynx:radiotherapy vs transoral robotic surgery, *QOL* quality of life, *EORTC* European organization for research and treatment of cancer, *HNCG-ROG* head and neck cancer group- radiation oncology group, *ECE* extracapsular extension, *PNI* perineural invasion, *LVI* lymphovascular invasion, *OS* overall survival, *RTOG* radiation therapy oncology group, *SIRS* Sinai robotic surgery trial, *TROG* trans-tasman radiation oncology group

### Treatment-related morbidity considerations

When no single modality emerges as notably superior in terms of survival/control outcomes, comparable treatment morbidity is scrutinized. There are some unique treatment-related morbidities to consider with regards to surgery such as bleeding. Bleeding is commonly reported as less than 5 %, though even in experienced hands, bleeding may occur in over 10 % of cases [57–60]. In a survey of over 2000 TORS procedures performed by 45 surgeons, there were 6 deaths (0.3 %) reported secondary to postoperative hemorrhage [60].

Regarding dysphagia in transoral surgery, most patients resume diet on the first postoperative day and long term feeding tube use is typically less than 10 % [48, 59, 60]. The need for long term feeding tube placement is associated with increase in T stage, location (tongue base vs tonsil), and extent of postoperative adjuvant therapy [61, 62]. Functional outcomes and quality of life are thought to be quite similar when comparing surgical resection with postoperative radiation and chemoradiation [63, 64].

### Anatomic considerations

While technical innovation in minimally invasive surgery has allowed transoral resection of a wide variety of tumors in many different locations, the occasional patient’s anatomy may preclude adequate exposure. In these scenarios transoral options may be ill advised, and consequently non-surgical management may be a preferable treatment approach. Rich et al. noted several relative (patient-related) contraindications: trismus, tongue bulk, limited neck extension, prominent teeth, mandibular tori, and limited transverse mandible dimension [6]. Retropharyngeal (or aberrant) internal carotid artery also poses a relative surgical contraindication. Moore et al. noted several relative (tumor-related) contraindications: deep extrinsic tongue muscle, mandible/skull base invasion, lateral neck, extensive laryngeal invasion, invasion of more than half base of tongue [53].

Given the anatomic restraints of the oropharynx, anticipation of adequate margin and subsequent defect is crucial. The surgeon needs to critically analyze the tumor as it relates to the surrounding anatomy and the likelihood of obtaining a negative margin, as close or positive margins may prompt additional therapeutic modalities in order to obtain adequate disease control, and along with this will come additional treatment related side effects/morbidity.

### Cost considerations

Fundamental difficulties exist when attempting to review previous literature regarding comparative cost of treatment modality. For example, variability exists in what may be considered associated with the actual cost of care and reimbursement schedules vary widely. In addition, fixed equipment costs and secondary downstream costs such as lost wages may also be taken into consideration further complicating comparative analysis.

Moore et al., note that 25 % of all OPSCCA patients may be treated with surgery alone, while 75 % will require adjuvant therapy when applying NCCN criteria [65, 66]. Up-front surgical resection of OPSCCA followed by pathology-dictated post-operative adjuvant therapy according to NCCN guidelines was noted to be 5.29×’s (non-government) and 3.66×’s (government) less expensive than chemoradiation [67]. Interestingly, when comparing surgery vs non-surgical modalities for HPV-associated OPSCCA with N2 neck disease, in nearly all assumptions chemoradiation was considered the cost-effective therapy based on quality of life adjusted years divided by total costs; though if the loco-regional recurrence of TORS was able to be hypothetically reduced by 30–50 %, surgery would become the higher value treatment [68]. These findings further support additional prospective comparative analysis.

## Clinical considerations for the patient with early stage disease

Excellent prognosis typically accompanies early stage low tumor burden HPV-associated OPSCCA (T1-2 N0-1 M0), and optimal therapy is single modality, commonly with surgery or radiotherapy. Decision for the surgical option over radiation in this scenario largely depends on location and surgical accessibility of the primary. Lateralized tonsil and tongue base will largely do very well functionally with transoral surgery and ipsilateral selective neck dissection. In the context of a large T2 endophytic primary in the midline of the tongue base that will also require bilateral neck dissection, the decision of surgery vs radiotherapy may be somewhat more difficult given the greater associated surgical morbidity and long-term functional consequences. Nevertheless, in the absence of adverse pathologic features (positive margins, extracapsular spread), the patient will be able to avoid adjuvant radiation. Alternatively, radiation monotherapy to primary and ipsilateral/bilateral necks (based on primary location) may be used, reserving systemic therapy only for T2N1 (as a category 2B recommendation), or in the context of adverse features or positive margin (category 1 recommendation). Given the high rates of disease control for early stage disease, surgery should be recommended only if the odds of requiring adjuvant radiation are anticipated to be low.

## Clinical considerations for the patient with locally advanced resectable disease

Many of HPV-associated OPSCCA are noted to possess cervical metastasis on initial presentation, and consequently staged as III or IV. This group will require multimodality therapy, with the exception of favorable T1-2 N1 (as noted above). Outside the context of clinical trial, there are three main treatment options (1 surgical, 2 non-surgical): (1) surgical resection of the primary with ipsilateral/bilateral neck dissection with appropriate adjuvant therapy (radiation, chemoradiation) based on pathologic/nodal features; (2) concurrent chemoradiotherapy, saving surgery for salvage; and (3) induction chemotherapy followed by radiation vs chemoradiation. The use of induction chemotherapy is controversial (category 3 recommendation) and both PARADIGM and DeCIDE trials have failed to demonstrate a survival advantage [69, 70]. As a result, we will not expand on this potential option further in this setting.

One of the first considerations with surgery in locally advanced resectable disease is the ability to obtain adequate and negative margins, as failure to do so will ultimately likely require postoperative chemoradiation. But the ability to clear mucosal margins must be balanced with morbidity and functional consequence of resection. Higher T stage tumors have higher postoperative functional morbidity, particularly those involving the tongue base. When a sound oncologic resection will have significant morbidity, it is appropriate to consider chemoradiation the preferred alterative.

Regarding management of advanced neck disease, the possibility of extracapsular extension must be considered as based on current guidelines the patient will likely warrant chemotherapy in addition to postoperative radiation if extracapsular extension is found on pathologic review of the neck dissection specimen. A patient with negative margins on the primary with extracapsular extension in the neck is a suitable candidate for the ADEPT trial (interventional phase III trial for p16 positive OPSCCA patients treated initially with surgery and have extracapsular spread in the lymph nodes, randomized to receive radiation alone vs radiation plus cisplatin) though if not available, one may argue for concurrent chemoradiation for such a patient over surgery with post-operative chemoradiation. Patients with evidence of gross extracapsular extension of cancer metastases on pretreatment imaging studies should be offered primary chemoradiation.

## Clinical considerations for the unknown primary patient

In the context of unknown primary tumor, several reports have claimed an identification rate of between 75–90 % when removing both palatine and lingual tonsil beds [71–74]. In some situations, excision of the tonsil beds and neck dissection will be curative.

Low recurrence rates generally characterize this population. Consequently, the elective, comprehensive treatment of mucosal sites and contralateral neck may represent over-treatment for some of these patients. Galloway provides an excellent algorithm for decision-making for the unknown primary [75]. T0N1 treatment should involve single modality therapy, commonly neck dissection. T0N2a management is subdivided by risk profile; with lowest risk (p16+, less than 10 pack year smoker) patients receiving single modality therapy. T0N2a with greater than 10 pack year history will be treated with surgery plus radiation. In the case of T0N2a with radiographic extranodal extension, primary chemoradiation is the recommendation. T0N2b treatment is dictated by institutional philosophy and may be either surgery followed by radiation, vs primary chemoradiation, although surgery may be associated with less long-term toxicity. T0N2c and T0N3 are both recommended to be treated with primary chemoradiation. What constitutes adequate mucosal sampling (unilateral versus bilateral tonsillectomy, base of tongue mucusectomy) remains an area of controversy.

## Conclusion

The management of HPV-associated OPSCCA will continue to evolve and will likely change dramatically in the future, particularly with respect to de-intensification. The role of surgery for HPV-associated OPSCCA will continue to be defined and refined as treatments aim to minimize long-term functional consequences while maintaining disease control. To date, no treatment modality has emerged superior for HPV-associated OPSCCA, so interventions should consider unique characteristics of the tumor presentation in addition to patient preferences in order to optimize outcomes and quality of life given the prolonged survival typically associated with this unique disease. As a result, patient enrollment in clinical trials is of critical importance, as it provides opportunity to expand knowledge base, to find answers to important treatment-related questions, and offer de-intensification options to patients.

## Abbreviations

HNC, head and neck cancer; HPV, human papilloma virus; IMRT, intensity modulated radiation therapy; OPSCCA, oropharyngeal squamous cell carcinoma; TLM, transoral laser microsurgery; TORS, transoral robotic surgery

## References

[1] Siegel R, Naishadham D, Jemal A (2013). Cancer statistics, 2013. CA Cancer J Clin.

[2] Chaturvedi AK, Anderson WF, Lortet-Tieulent J (2013). Worldwide trends in incidence rates for oral cavity and oropharyngeal cancers. J Clin Oncol.

[3] Ang KK, Harris J, Wheeler R (2010). Human papillomavirus and survival of patients with oropharyngeal cancer. N Engl J Med.

[4] Fakhry C, Westra WH, Li S (2008). Improved survival of patients with human papillomavirus-positive head and neck squamous cell carcinoma in a prospective clinical trial. J Natl Cancer Inst.

[5] Licitra L, Perrone F, Bossi P (2006). High-risk human papillomavirus affects prognosis in patients with surgically treated oropharyngeal squamous cell carcinoma. J Clin Oncol.

[6] Rich JT, Milov S, Lewis JS, Thorstad WL, Adkins DR, Haughey BH (2009). Transoral laser microsurgery (TLM) +/- adjuvant therapy for advanced stage oropharyngeal cancer: outcomes and prognostic factors. Laryngoscope.

[7] Mehanna H, Beech T, Nicholson T (2013). Prevalence of human papillomavirus in oropharyngeal and nonoropharyngeal head and neck cancer--systematic review and meta-analysis of trends by time and region. Head Neck.

[8] Chaturvedi AK, Engels EA, Pfeiffer RM (2011). Human papillomavirus and rising oropharyngeal cancer incidence in the United States. J Clin Oncol.

[9] Rischin D, Young RJ, Fisher R (2010). Prognostic significance of p16INK4A and human papillomavirus in patients with oropharyngeal cancer treated on TROG 02.02 phase III trial. J Clin Oncol.

[10] Dayyani F, Etzel CJ, Liu M, Ho CH, Lippman SM, Tsao AS (2010). Meta-analysis of the impact of human papillomavirus (HPV) on cancer risk and overall survival in head and neck squamous cell carcinomas (HNSCC). Head Neck Oncol.

[11] Heiduschka G, Grah A, Oberndorfer F (2015). Improved survival in HPV/p16-positive oropharyngeal cancer patients treated with postoperative radiotherapy. Strahlenther Onkol.

[12] Andl T, Kahn T, Pfuhl A (1998). Etiological involvement of oncogenic human papillomavirus in tonsillar squamous cell carcinomas lacking retinoblastoma cell cycle control. Cancer Res.

[13] Lindel K, Beer KT, Laissue J, Greiner RH, Aebersold DM (2001). Human papillomavirus positive squamous cell carcinoma of the oropharynx: a radiosensitive subgroup of head and neck carcinoma. Cancer.

[14] Ragin CC, Taioli E (2007). Survival of squamous cell carcinoma of the head and neck in relation to human papillomavirus infection: review and meta-analysis. Int J Cancer.

[15] Fakhry C, Zhang Q, Nguyen-Tan PF (2014). Human papillomavirus and overall survival after progression of oropharyngeal squamous cell carcinoma. J Clin Oncol.

[16] Fischer CA, Kampmann M, Zlobec I (2010). p16 expression in oropharyngeal cancer: its impact on staging and prognosis compared with the conventional clinical staging parameters. Ann Oncol.

[17] Hafkamp HC, Manni JJ, Haesevoets A (2008). Marked differences in survival rate between smokers and nonsmokers with HPV 16-associated tonsillar carcinomas. Int J Cancer.

[18] O’Sullivan B, Huang SH, Su J (2016). Development and validation of a staging system for HPV-related oropharyngeal cancer by the International Collaboration on Oropharyngeal cancer Network for Staging (ICON-S): a multicentre cohort study. Lancet Oncol.

[19] Fakhry C, Gillison ML (2006). Clinical implications of human papillomavirus in head and neck cancers. J Clin Oncol.

[20] Brockstein BE, Vokes EE (2011). Head and neck cancer in 2010: Maximizing survival and minimizing toxicity. Nat Rev Clin Oncol.

[21] Givens DJ, Karnell LH, Gupta AK (2009). Adverse events associated with concurrent chemoradiation therapy in patients with head and neck cancer. Arch Otolaryngol Head Neck Surg.

[22] Maxwell JH, Mehta V, Wang H (2014). Quality of life in head and neck cancer patients: impact of HPV and primary treatment modality. Laryngoscope.

[23] Hunter KU, Schipper M, Feng FY (2013). Toxicities affecting quality of life after chemo-IMRT of oropharyngeal cancer: prospective study of patient-reported, observer-rated, and objective outcomes. Int J Radiat Oncol Biol Phys.

[24] de Almeida JR, Byrd JK, Wu R (2014). A systematic review of transoral robotic surgery and radiotherapy for early oropharynx cancer: a systematic review. Laryngoscope.

[25] Al-Mamgani A, van Rooij P, Verduijn GM, Mehilal R, Kerrebijn JD, Levendag PC (2013). The impact of treatment modality and radiation technique on outcomes and toxicity of patients with locally advanced oropharyngeal cancer. Laryngoscope.

[26] Beitler JJ, Zhang Q, Fu KK (2014). Final results of local-regional control and late toxicity of RTOG 9003: a randomized trial of altered fractionation radiation for locally advanced head and neck cancer. Int J Radiat Oncol Biol Phys.

[27] Nguyen-Tan PF, Zhang Q, Ang KK (2014). Randomized phase III trial to test accelerated versus standard fractionation in combination with concurrent cisplatin for head and neck carcinomas in the Radiation Therapy Oncology Group 0129 trial: long-term report of efficacy and toxicity. J Clin Oncol.

[28] Woody NM, Koyfman SA, Xia P (2016). Regional control is preserved after dose de-escalated radiotherapy to involved lymph nodes in HPV positive oropharyngeal cancer. Oral Oncol.

[29] Cmelak A, Li S, Marur S (2014). ECOG 1308: A phase II trial of induction chemotherapy followed by cetuximab with low dose versus standard dose IMRT in patients with HPV-associated resectable squamous cell carcinoma of the oropharynx. J Clin Oncol.

[30] Adelstein DJ, Li Y, Adams GL (2003). An intergroup phase III comparison of standard radiation therapy and two schedules of concurrent chemoradiotherapy in patients with unresectable squamous cell head and neck cancer. J Clin Oncol.

[31] Bonner JA, Harari PM, Giralt J (2010). Radiotherapy plus cetuximab for locoregionally advanced head and neck cancer: 5-year survival data from a phase 3 randomised trial, and relation between cetuximab-induced rash and survival. Lancet Oncol.

[32] Blanchard P, Baujat B, Holostenco V (2011). Meta-analysis of chemotherapy in head and neck cancer (MACH-NC): a comprehensive analysis by tumour site. Radiother Oncol.

[33] Pignon JP, le Maitre A, Bourhis J, Group M-NC (2007). Meta-Analyses of Chemotherapy in Head and Neck Cancer (MACH-NC): an update. Int J Radiat Oncol Biol Phys.

[34] Cooper JS, Zhang Q, Pajak TF (2012). Long-term follow-up of the RTOG 9501/intergroup phase III trial: postoperative concurrent radiation therapy and chemotherapy in high-risk squamous cell carcinoma of the head and neck. Int J Radiat Oncol Biol Phys.

[35] Bernier J, Cooper JS, Pajak TF (2005). Defining risk levels in locally advanced head and neck cancers: a comparative analysis of concurrent postoperative radiation plus chemotherapy trials of the EORTC (#22931) and RTOG (# 9501). Head Neck.

[36] Maxwell JH, Ferris RL, Gooding W (2013). Extracapsular spread in head and neck carcinoma: impact of site and human papillomavirus status. Cancer.

[37] Lewis JS, Carpenter DH, Thorstad WL, Zhang Q, Haughey BH (2011). Extracapsular extension is a poor predictor of disease recurrence in surgically treated oropharyngeal squamous cell carcinoma. Mod Pathol.

[38] Sinha P, Lewis JS, Piccirillo JF, Kallogjeri D, Haughey BH (2012). Extracapsular spread and adjuvant therapy in human papillomavirus-related, p16-positive oropharyngeal carcinoma. Cancer.

[39] Calais G, Bardet E, Sire C (2004). Radiotherapy with concomitant weekly docetaxel for Stages III/IV oropharynx carcinoma. Results of the 98-02 GORTEC Phase II trial. Int J Radiat Oncol Biol Phys.

[40] Machtay M, Moughan J, Trotti A (2008). Factors associated with severe late toxicity after concurrent chemoradiation for locally advanced head and neck cancer: an RTOG analysis. J Clin Oncol.

[41] Cohen MA, Weinstein GS, O’Malley BW, Feldman M, Quon H (2011). Transoral robotic surgery and human papillomavirus status: Oncologic results. Head Neck.

[42] Genden EM, Kotz T, Tong CC (2011). Transoral robotic resection and reconstruction for head and neck cancer. Laryngoscope.

[43] White HN, Moore EJ, Rosenthal EL (2010). Transoral robotic-assisted surgery for head and neck squamous cell carcinoma: one- and 2-year survival analysis. Arch Otolaryngol Head Neck Surg.

[44] Moore EJ, Olsen SM, Laborde RR (2012). Long-term functional and oncologic results of transoral robotic surgery for oropharyngeal squamous cell carcinoma. Mayo Clin Proc.

[45] Choby GW, Kim J, Ling DC (2015). Transoral robotic surgery alone for oropharyngeal cancer: quality-of-life outcomes. JAMA Otolaryngol Head Neck Surg.

[46] Dowthwaite SA, Franklin JH, Palma DA, Fung K, Yoo J, Nichols AC (2012). The role of transoral robotic surgery in the management of oropharyngeal cancer: a review of the literature. ISRN Oncol.

[47] Steiner W, Fierek O, Ambrosch P, Hommerich CP, Kron M (2003). Transoral laser microsurgery for squamous cell carcinoma of the base of the tongue. Arch Otolaryngol Head Neck Surg.

[48] Haughey BH, Hinni ML, Salassa JR (2011). Transoral laser microsurgery as primary treatment for advanced-stage oropharyngeal cancer: a United States multicenter study. Head Neck.

[49] Quon H, Richmon JD (2012). Treatment deintensification strategies for HPV-associated head and neck carcinomas. Otolaryngol Clin N Am.

[50] Walvekar RR, Li RJ, Gooding WE (2008). Role of surgery in limited (T1-2, N0-1) cancers of the oropharynx. Laryngoscope.

[51] Posner MR, Lorch JH, Goloubeva O (2011). Survival and human papillomavirus in oropharynx cancer in TAX 324: a subset analysis from an international phase III trial. Ann Oncol.

[52] Corry J, Peters LJ, Rischin D (2015). Impact of center size and experience on outcomes in head and neck cancer. J Clin Oncol.

[53] Moore EJ, Hinni ML (2013). Critical review: transoral laser microsurgery and robotic-assisted surgery for oropharynx cancer including human papillomavirus-related cancer. Int J Radiat Oncol Biol Phys.

[54] Rinaldi V, Pagani D, Torretta S, Pignataro L (2013). Transoral robotic surgery in the management of head and neck tumours. Ecancermedicalscience.

[55] Gillison ML, Zhang Q, Jordan R R (2012). Tobacco smoking and increased risk of death and progression for patients with p16-positive and p16-negative oropharyngeal cancer. J Clin Oncol.

[56] Anantharaman D, Muller DC, Lagiou P, et al. Combined effects of smoking and HPV16 in oropharyngeal cancer. Int J Epidemiol. 2016. 10.1093/ije/dyw069 PMC5841602 27197530

[57] Canis M, Martin A, Kron M (2013). Results of transoral laser microsurgery in 102 patients with squamous cell carcinoma of the tonsil. Eur Arch Oto-rhino-laryngol.

[58] Pollei TR, Hinni ML, Moore EJ (2013). Analysis of postoperative bleeding and risk factors in transoral surgery of the oropharynx. JAMA Otolaryngol Head Neck Surg.

[59] Weinstein GS, O’Malley BW (2012). Magnuson JSet al. Transoral robotic surgery: a multicenter study to assess feasibility, safety, and surgical margins. Laryngoscope.

[60] Chia SH, Gross ND, Richmon JD (2013). Surgeon experience and complications with Transoral Robotic Surgery (TORS). Otolaryngol Head Neck Surg.

[61] Sinclair CF, McColloch NL, Carroll WR, Rosenthal EL, Desmond RA, Magnuson JS (2011). Patient-perceived and objective functional outcomes following transoral robotic surgery for early oropharyngeal carcinoma. Arch Otolaryngol Head Neck Surg.

[62] Dziegielewski PT, Teknos TN, Durmus K (2013). Transoral robotic surgery for oropharyngeal cancer: long-term quality of life and functional outcomes. JAMA Otolaryngol Head Neck Surg.

[63] More YI, Tsue TT, Girod DA (2013). Functional swallowing outcomes following transoral robotic surgery vs primary chemoradiotherapy in patients with advanced-stage oropharynx and supraglottis cancers. JAMA Otolaryngol Head Neck Surg.

[64] Chen AM, Daly ME, Luu Q, Donald PJ, Farwell DG (2015). Comparison of functional outcomes and quality of life between transoral surgery and definitive chemoradiotherapy for oropharyngeal cancer. Head Neck.

[65] Moore EJ, Henstrom DK, Olsen KD, Kasperbauer JL, McGree ME (2009). Transoral resection of tonsillar squamous cell carcinoma. Laryngoscope.

[66] Moore EJ, Olsen KD, Kasperbauer JL (2009). Transoral robotic surgery for oropharyngeal squamous cell carcinoma: a prospective study of feasibility and functional outcomes. Laryngoscope.

[67] Moore EJ, Hinni ML, Olsen KD, Price DL, Laborde RR, Inman JC (2012). Cost considerations in the treatment of oropharyngeal squamous cell carcinoma. Otolaryngol Head Neck Surg.

[68] Sher DJ, Fidler MJ, Tishler RB, Stenson K, Al-Khudari S (2016). Cost-Effectiveness Analysis of Chemoradiation Therapy Versus Transoral Robotic Surgery for Human Papillomavirus-Associated, Clinical N2 Oropharyngeal Cancer. Int J Radiat Oncol Biol Phys.

[69] Haddad R, O’Neill A, Rabinowits G (2013). Induction chemotherapy followed by concurrent chemoradiotherapy (sequential chemoradiotherapy) versus concurrent chemoradiotherapy alone in locally advanced head and neck cancer (PARADIGM): a randomised phase 3 trial. Lancet Oncol.

[70] Cohen EE, Karrison TG, Kocherginsky M (2014). Phase III randomized trial of induction chemotherapy in patients with N2 or N3 locally advanced head and neck cancer. J Clin Oncol.

[71] Durmus K, Rangarajan SV, Old MO, Agrawal A, Teknos TN, Ozer E (2014). Transoral robotic approach to carcinoma of unknown primary. Head Neck.

[72] Graboyes EM, Sinha P, Thorstad WL, Rich JT, Haughey BH (2015). Management of human papillomavirus-related unknown primaries of the head and neck with a transoral surgical approach. Head Neck.

[73] Mehta V, Johnson P, Tassler A (2013). A new paradigm for the diagnosis and management of unknown primary tumors of the head and neck: a role for transoral robotic surgery. Laryngoscope.

[74] Patel SA, Magnuson JS, Holsinger FC (2013). Robotic surgery for primary head and neck squamous cell carcinoma of unknown site. JAMA Otolaryngol Head Neck Surg.

[75] Galloway TJ, Ridge JA (2015). Management of Squamous Cancer Metastatic to Cervical Nodes With an Unknown Primary Site. J Clin Oncol.

